# Identification of common genetic factors and immune-related pathways associating more than two autoimmune disorders: implications on risk, diagnosis, and treatment

**DOI:** 10.1186/s44342-024-00004-5

**Published:** 2024-07-02

**Authors:** Aruna Rajalingam, Anjali Ganjiwale

**Affiliations:** Department of Life Science, Bangalore University, Bangalore, Karnataka 560056 India

**Keywords:** Autoimmune disorders (ADs), Immunological tolerance, Genetic factors, Biomarkers, Differential gene expression, Immune-related pathways

## Abstract

Autoimmune disorders (ADs) are chronic conditions resulting from failure or breakdown of immunological tolerance, resulting in the host immune system attacking its cells or tissues. Recent studies report shared effects, mechanisms, and evolutionary origins among ADs; however, the possible factors connecting them are unknown. This study attempts to identify gene signatures commonly shared between different autoimmune disorders and elucidate their molecular pathways linking the pathogenesis of these ADs using an integrated gene expression approach. We employed differential gene expression analysis across 19 datasets of whole blood/peripheral blood cell samples with five different autoimmune disorders (rheumatoid arthritis, multiple sclerosis, systemic lupus erythematosus, Crohn’s disease, and type 1 diabetes) to get nine key genes—EGR1, RUNX3, SMAD7, NAMPT, S100A9, S100A8, CYBB, GATA2, and MCEMP1 that were primarily involved in cell and leukocyte activation, leukocyte mediated immunity, IL-17, AGE-RAGE signaling in diabetic complications, prion disease, and NOD-like receptor signaling confirming its role in immune-related pathways. Combined with biological interpretations such as gene ontology (GO), pathway enrichment, and protein–protein interaction (PPI) network, our current study sheds light on the in-depth research on early detection, diagnosis, and prognosis of different ADs.

**Supplementary Information:**

The online version contains supplementary material available at 10.1186/s44342-024-00004-5.

## Introduction

The function of the immune system is to protect the host from the attack of any foreign particle invading the cell. The entry of foreign particles activates a two-step immune response that triggers innate immunity and then converts to the adaptive or specific immune response. During this process, our immune system should be capable of recognizing self and non-self-antigens, failure of which results in the breakdown of self-tolerance, leading to the development of autoimmune disease [1]. The incidence and prevalence of autoimmune diseases are increasing rapidly, affecting more than 5% of the population worldwide with increased morbidity and mortality rates [2]. Literature sources report that these disorders are common and steadily growing in westernized societies [3]. The pathogenesis of autoimmune diseases has been challenging and unknown due to various etiological factors. Several factors such as genetics, environmental factors, sex, and race are thought to contribute to the development of an immune response [4, 5]. Human leukocyte antigen (HLA) genes at the short arm of chromosome number 6 have been reported to have a strong linkage with rheumatoid arthritis (RA) along with PTPN22 (protein tyrosine phosphatase non-receptor type 22), PADI4 (peptidyl arginine deiminase 4), STAT4 (signal transducer and activator of transcription 4), and RUN X1 (runt-related transcription factor 1) [6]. Systemic lupus erythematosus (SLE) genes are associated with the components of the complement activation pathway, IgG Fc receptors (immunoglobulin Fc receptor), and HLA region. HLA gene clusters also contribute 20–60% of the risk of causing multiple sclerosis (MS) [7]. INS (insulin), PTPN22, PTPN2 (protein tyrosine phosphatase non-receptor 2), IL2RA (interleukin 2 (IL2) receptor alpha), CTLA4 (cytotoxic T lymphocyte antigen-4), and IFIH1 (interferon induced with helicase C domain 1) are some non-major histocompatibility (MHC) genes associated with type 1 diabetes mellitus (T1D) [8]. Environmental factors such as infectious agents, ultraviolet light, chemicals, environmental pollutants, smoking, and diet also help trigger autoimmune responses [9–13]. In addition, DNA and RNA viral infections with mechanisms such as molecular mimicry, protein changes, or the exposition of cryptic antigens [14–17] are also known to induce autoimmune disorders [18]. Present treatment for autoimmune disease aims to reduce chronic symptoms by lowering the level of immune system activity that makes patients often face a lifetime of debilitating symptoms, loss of organ and tissue function, and high medical costs. However, treatments vary widely depending on the disease and the symptoms [19].

Several studies have reported that there is an association among autoimmune disorders. However, the possible factors connecting them have yet to be addressed completely. Kimura and his colleagues have reported that the observed prevalence of MS at the onset of inflammatory bowel disease (IBD) was 3.7 times greater than the expected prevalence. This study confirms an association between 2 diseases (MS and IBD). However, they failed to speculate on possible mechanisms in this association [20]. In 2010, Solomon and his colleagues reported an increased risk of developing diabetes mellitus (DM) in RA, psoriatic arthritis, or psoriasis (PsA/PsO) patients. Later, in 2017, a survey on RA patients revealed that out of 2535 RA patients, 20% had developed DM [21]. Psoriasis (Ps) is another autoimmune disorder linked with increased C-reactive protein (CRP) in Ps patients. These increased CRP levels correlate with increased serum concentrations of IL-6 (interleukin-6), S100A8, and S100A9 proteins. The CRP level concentration is also high in psoriatic arthritis (PsA) patients, forming atherosclerotic plaques [22]. Obesity, a feature of metabolic syndrome, was reported in Ps and PsA patients. So, there is an association between obesity, Ps, and PsA [22–25]. Functional polymorphism on CD40 (cluster of differentiation 40) is associated with CD and MS sharing a common signaling pathway between these two autoimmune disorders [26]. Also, IL23R (interleukin-23 receptor) gene polymorphism is associated with inflammatory bowel disease, psoriasis, and ankylosing spondylitis [27]. Differential expression of the CDKAL1 (Cdk5 regulatory associated protein 1-like 1) gene is reported in psoriasis. It is also implicated in the pathogenesis of Crohn’s disease (CD) and type II diabetes (TIID) [28]. Thus, literature studies answer the “common genetic origin” theory of autoimmune disorders. Association and linkage studies in different populations have also revealed that several susceptibility loci overlap in ADs, and clinical studies have shown the frequent clustering of several ADs [29]. A recent study in 2023 also confirms that there are shared effects, mechanisms, and evolutionary origins among ADs [30]. So, identifying shared genetic factors helps us better understand their pathogenesis mechanism which controls the onset, severity, and chronicity of the disorders [31].

This study attempts to identify genetic factors shared between five autoimmune disorders and elucidate their molecular pathways linking their pathogenesis mechanism. We conducted a combined transcriptome analysis of five autoimmune disorders (Rheumatoid arthritis, multiple sclerosis, systemic lupus erythematosus, Crohn’s disease, and type 1 diabetes) of whole blood/peripheral blood tissue samples with an integrated gene expression approach to identify essential gene markers contributing to the pathophysiology of autoimmune disorders from the existing gene expression data retrieved from the Gene Expression Omnibus (GEO) database. After eliminating low sample size (less than 10) datasets of autoimmune disorders, our approach involves the identification of differentially expressed genes (DEGs) to filter out the most significant shared gene signature between healthy and autoimmune disorder samples to derive insightful inferences on biological functions and pathways involved.

## Materials and methods

### Data collection

All microarray datasets were obtained from the Gene Expression Omnibus database (GEO, https://www.ncbi.nlm.nih.gov/geo/) [32]. The GEO database for microarray datasets was filtered using the keywords “Rheumatoid arthritis (RA),” “Multiple sclerosis (MS),” “Systemic lupus erythematosus (SLE),” “Crohn’s disease (CD),” and “Type 1 diabetes mellitus (T1D).” Datasets were downloaded with Whole blood/Peripheral blood cells and *Homo sapiens* as inclusion criteria. Nineteen microarray datasets were qualified to be selected. Samples less than ten were excluded for analysis, resulting in 16 gene expression microarray datasets.

GEO expression datasets for RA included GSE93272, GSE15573, and GSE17755 (175 disease and 103 control samples); MS included GSE17048, GSE21942, GSE26484, and GSE141804 (134 disease and 74 control samples); SLE included GSE17755, GSE30153, GSE81622, and GSE72326 (226 disease and 107 control samples); T1D included GSE11907 and GSE142153 (43 disease and 26 control samples); and CD included GSE119600, GSE26124, and GSE3365 (193 disease and 121 control samples), respectively. A total of 1202 samples were taken for the study.

### Differentially expressed genes (DEGs) analysis by GEO2R tool

GEO2R analysis tool with the limma package from Bioconductor to display the *t*-test score, *p*-value, and adjusted *p*-score was used to analyze differentially expressed genes of all the individual 16 datasets (https://www.ncbi.nlm.nih.gov/geo/geo2r/). The feature reduction technique UMAP (Uniform Manifold Approximation and Projection), a part of the GEO2R script, was used for dimensionality reduction for all 16 datasets. The top 250 DEGs from each dataset were obtained by setting the significance level cut-off (*p* < 0.05), and their values were adjusted using Benjamini–Hochberg correction (false discovery rate, FDR) (Supplementary Information A1) [33].

The Venn diagram is the most common and apparent data visualization for illustrating the overlap and difference between data sets [34]. We used the Python Venn package (Version 0.11.7) to find the common DEGs between RA, MS, SLE, CD, and T1D datasets (https://github.com/tctianchi/pyvenn).

### Function enrichment analysis

To explore the enriched biological pathways and annotations in terms of gene ontology (GO), a graphical tool for gene set enrichment analysis—ShinyGO (Version 0.76) (http://bioinformatics.sdstate.edu/go/)—was used. It produces hierarchical clustering trees, networks summarizing overlapping pathways, and gene characteristics plots [35]. The Kyoto Encyclopedia of Genes and Genomes (KEGG) pathway enrichment analysis was performed using Database for Annotation, Visualization and Integrated Discovery (DAVID) v6.8 (https://david.abcc.ncifcrf.gov) [36]. The enriched pathways were defined based on the *p*-value < 0.05.

### Network topology-based analysis (NTA)

The WebGestalt (WEB-based Gene Set Analysis Toolkit) was used to compute and visualize the topological network properties of DEGs in a molecular interaction network [37]. Different topological characteristics, such as the centrality of nodes in the network, were compared with known GO pathways. All the results for the top 10 GO categories are reported with a statistical significance of *p*-value < 0.05. The protein–protein interactions (PPI) network of DEGs was established by using STRING (Version 11.5) (https://string-db.org) [38]. A gene–gene interaction network was performed for the significant DEGs and candidate hub genes were selected through the GeneMANIA Cytoscape plugin command line tool [39] by calculating the network weight that reflects the data source relevance for predicting the function of genes.

### Validation of candidate hub genes and their significance

The significance of the selected hub genes in contributing to various other disorders was analyzed using ToppGene Suite (http://toppgene.cchmc.org), a one-stop portal for gene list enrichment analysis and candidate gene prioritization based on functional annotation and protein interactions network [40].

### Chemical—gene interaction network analysis

The chemical gene/protein interactions, chemical diseases, and gene-disease relationships were analyzed using a comparative toxicological genomics database (CTD, http://ctdbase.org/) was used to analyze [41]. Diagnostic markers and the molecular compound interaction network were visualized using Cytoscape software.

## Results

### Identification of DEGs in RA, MS, SLE, CD, and T1D datasets

GEO expression datasets with a total of 1202 AD samples versus healthy controls are reported in Supplementary Table S1. The top 250 DEGs from each dataset were obtained by setting the significance level cut-off (*p* ≤ 0.05), and their values were adjusted using Benjamini–Hochberg correction (false discovery rate) using the GEO2R analysis tool. The analysis resulted in the DEGs that were ranked by adjusted *p*-value (*p*-values corrected for multiple testing). A total of 545 DEGs were identified across all RA datasets, 1027 DEGs were identified across all MS datasets, 701 DEGs were identified across all SLE datasets, 639 DEGs were identified across all T1D datasets, and 621 DEGs were identified across all CD datasets (Supplementary Table S2). These DEGs were further analyzed to identify significant genes in ADs.

### Identification of 32 DEGs

**Fig. 1:**
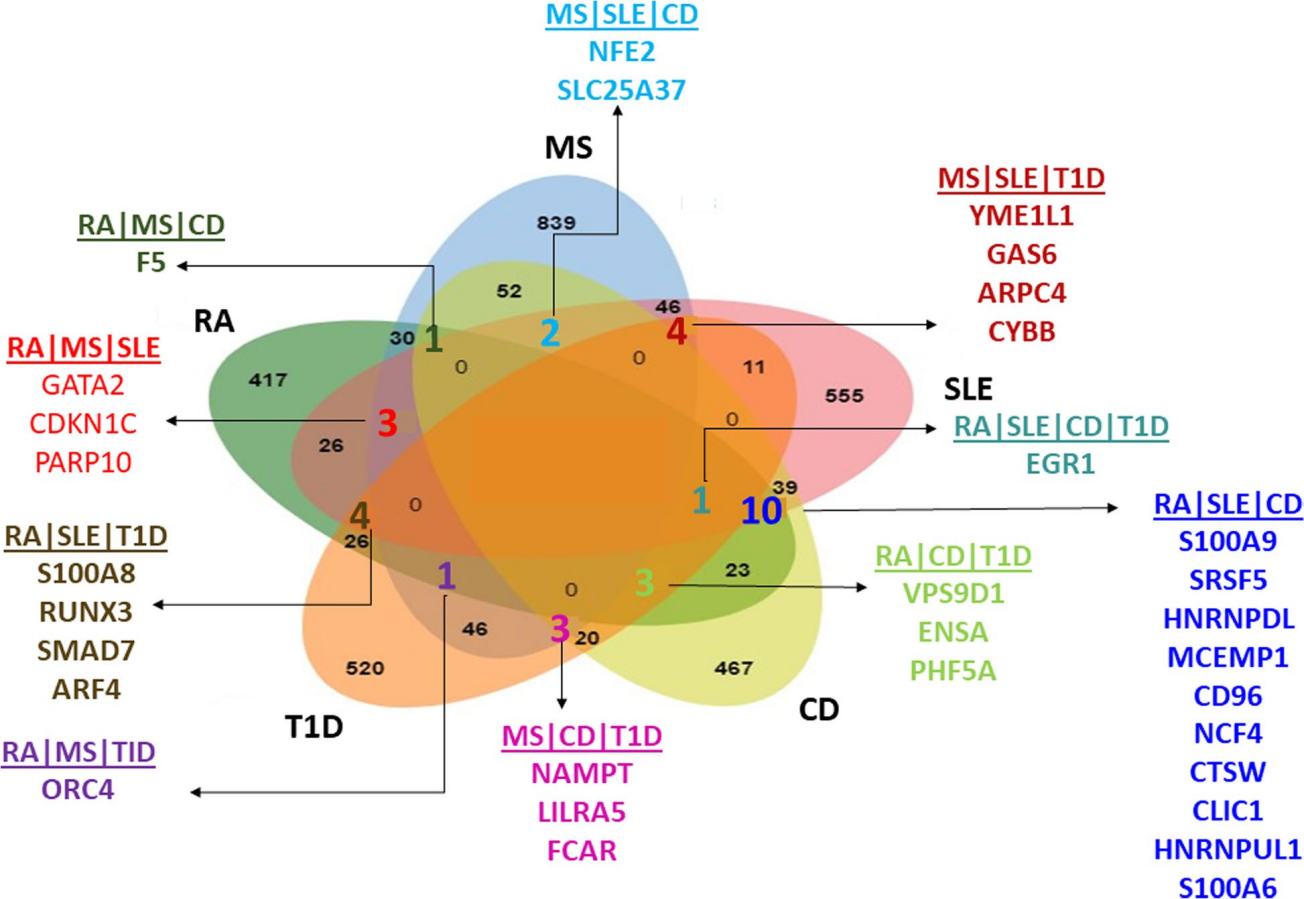
Identification of common DEGs between ADs. Significant genes (32 DEGs) expressed in more than 2 ADs are highlighted

When two AD datasets were compared, we found that 30, 26, 23, and 26 DEGs were shared by RA|MS, RA|SLE, RA|CD, and RA|T1D datasets. Forty-six, 52, and 46 DEGs were shared by the MS|SLE, MS|CD, and MS|T1D datasets. Thirty-nine DEGs were shared between SLE|CD, 11 DEGs were shared between SLE|T1D datasets, and 20 DEGs were common between CD|T1D datasets. When three AD datasets were compared, we found 3 DEGs (GATA2, CDKN1C, PARP10) are common in RA|MS|SLE, 1 gene (F5) common in RA|MS|CD, and 1 gene (ORC4) is common in RA|MS|T1D datasets. Ten genes (S100A9, SRSF5, HNRNPDL, MCEMP1, CD96, NCF4, CTSW, CLIC1, HNRNPUL1, S100A6) and four genes (S100A8, RUNX3, SMAD7, ARF4) had common DEGs in RA|SLE|CD and RA|SLE|T1D datasets. RA|CD|T1D datasets have three common genes (VPS9D1, ENSA, PHF5A). MS|SLE|CD and MS|SLE|T1D datasets share 2 genes (NFE2, SLC25A37) and 4 genes (YME1L1, GAS6, ARPC4, CYBB). MS|CD|T1D datasets share three genes (NAMPT, LILRA5, FCAR)**.** When comparing four datasets, we found that EGR1 is common in the RA, SLE, CD, and T1D datasets (Fig. 1 and Supplementary Table S3). However, there were no common genes among all 5 AD datasets. Thirty-two DEGs that were expressed in more than 2 ADs were identified as significant genes in AD pathogenesis (Supplementary Table S3).

### GO enrichment analysis of DEGs

GO enrichment analysis showed the involvement of significant genes in various biological processes (BP), molecular functions (MF), and cellular components (CC), respectively.

#### Biological processes (BP)

**Fig. 2:**
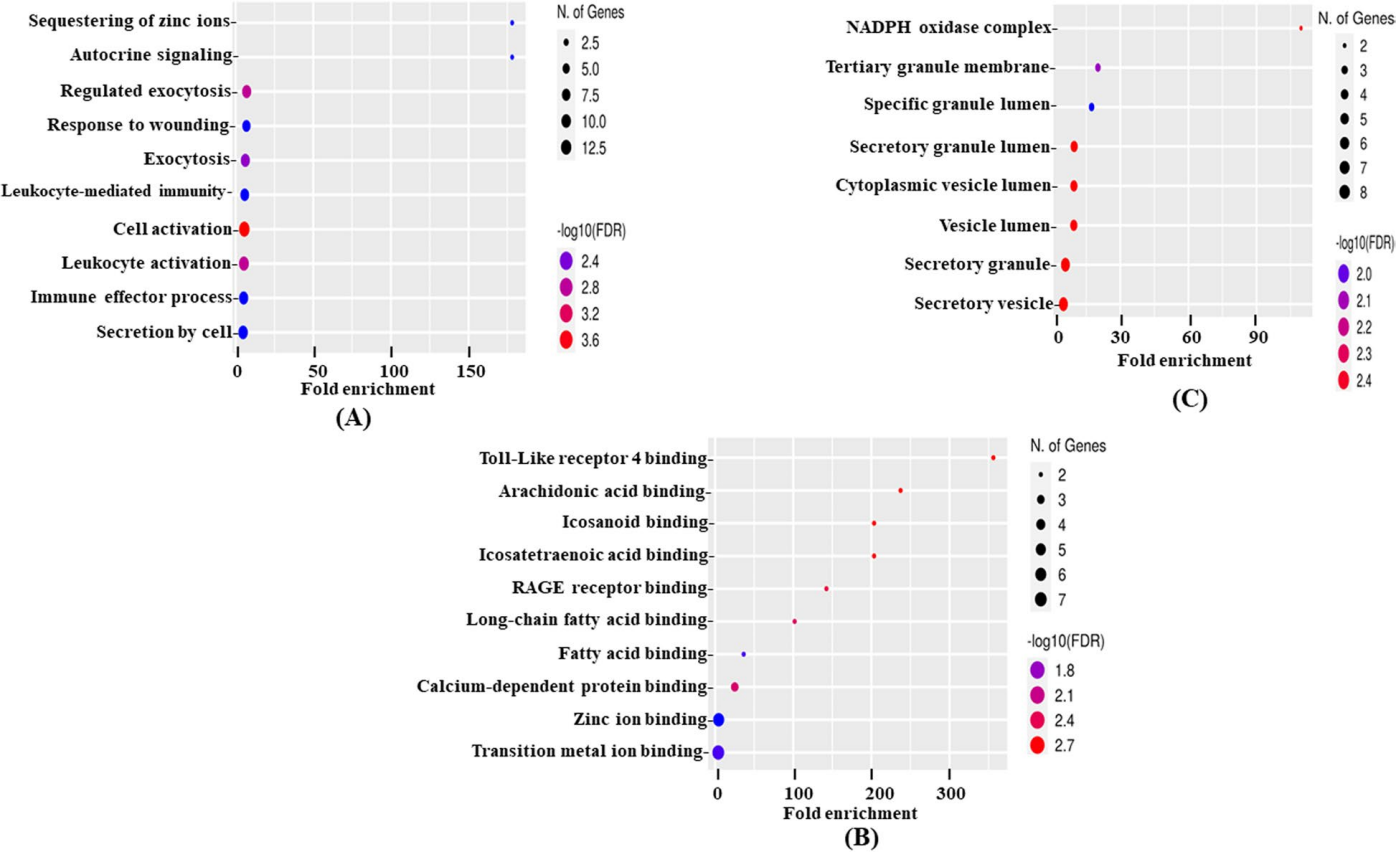
GO Enrichment analysis of 32 DEGs in **A** biological processes, **B** molecular functions, and **C** cellular components. Top pathways are sorted by fold enrichment enriched with significant DEGs. The dot size represents the count of differentially expressed genes, and the color depth represents the enrichment FDR. Detailed GO enrichment analysis has been provided in the supplemental information

**Fig. 3:**
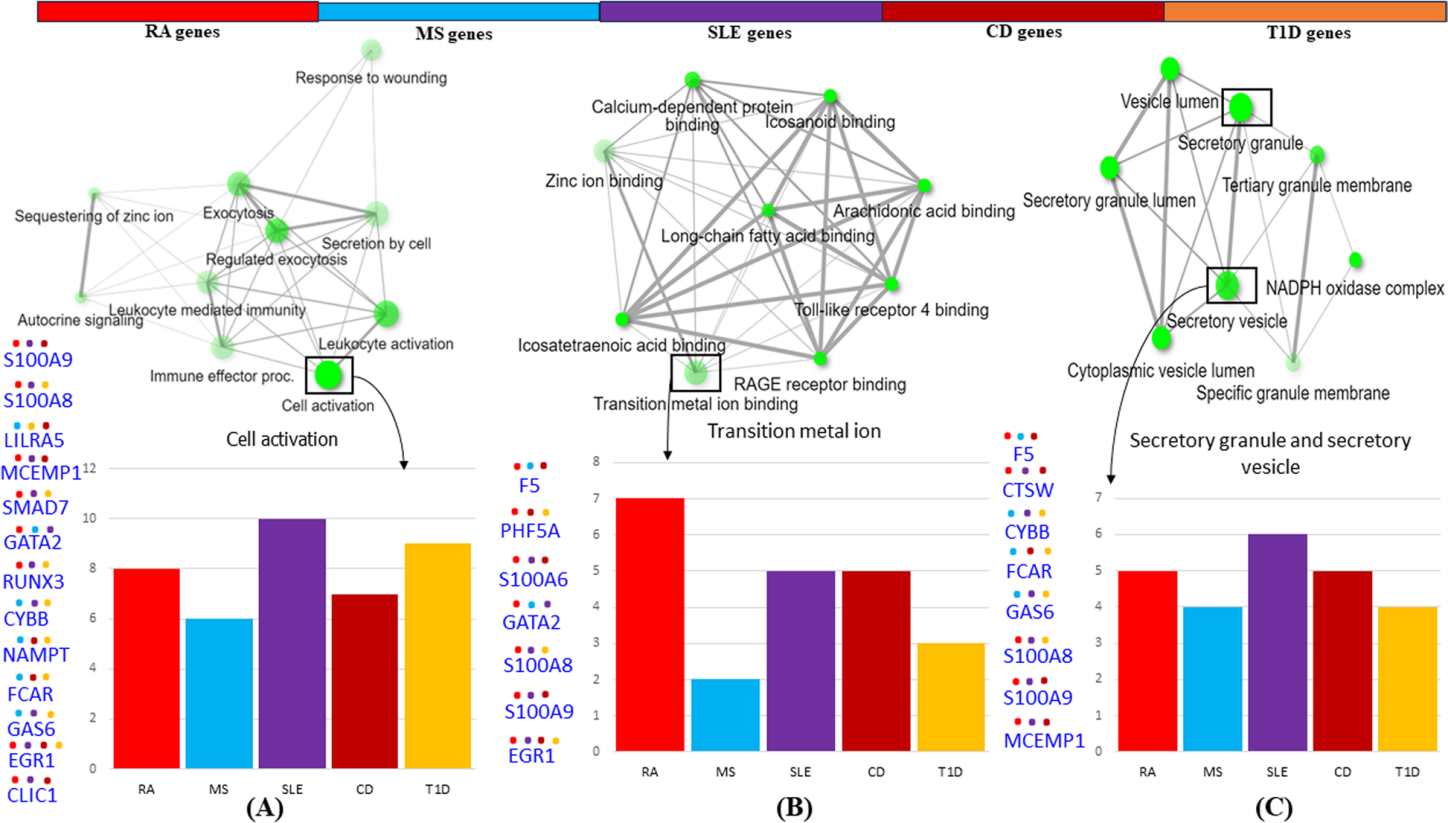
GO enrichment network of **A** biological processes, **B** molecular functions, and **C** cellular components are represented at edge cut-off 0.3. Each node represents an enriched GO term. Related GO terms are connected by a line, whose thickness reflects the percent of overlapping genes. The size of the node corresponds to the number of genes. Darker nodes represent significantly enriched gene sets. The bar plot and the genes in each ontology are color-coded with red (RA), blue (MS), purple (SLE), brown (CD), and orange (T1D). Significant pathways are highlighted in each ontology based on the larger gene sets. Detailed information has been provided in the supplemental information

In BP, significant DEGs were enriched in a total of 855 pathways (Supplementary Table S4), among which the top 10 pathways are cell activation, secretion by cell, leukocyte activation, immune effector process, exocytosis, leukocyte mediated immunity, regulated exocytosis, response to wounding, sequestering of ions, and autocrine signaling which is sorted based on fold enrichment (Fig. 2A). The hierarchical clustering tree explains the correlation among the significant pathways in which exocytosis, regulated exocytosis, autocrine signaling, and sequestering of zinc ions are depicted as redundant pathways. It also reveals that Leukocyte and cell activation pathways have many shared DEGs with significant *p*-values (Supplementary Table S5, Supplementary Fig. 1A). The network plot shows the relationship between the enrichment pathways in which each node (enriched GO pathways) is connected if at least 20% or more genes are shared. Leukocyte and cell activation pathways show large, overlapping, and significantly enriched gene sets. The bar plot shows the disease significance of BP ontology. Cell activation is considered a significant pathway based on larger gene sets involving a total of 13 genes, out of which 10 contribute to SLE pathogenesis (Supplementary Table S5, Fig. 3A). Supplementary Table S6 shows the significant genes grouped by functional categories defined by high-level GO terms. The DEGs are enriched mainly in the immune system’s processes of stress and regulation of biological process pathways.

#### Molecular functions (MF)

In MF, DEGs were enriched in 75 pathways (Supplementary Table S4), with the top pathways involved in transition metal ion binding, zinc ion binding, and calcium-dependent protein binding (Fig. 2B). A hierarchical clustering tree summarizes the correlation among the significant pathways, in which many shared genes are clustered together (Supplementary Table S5, Supplementary Fig. 1B). Transition metal ion binding and zinc ion binding pathways represent larger gene sets, with more overlap and enriched gene sets. The bar plot shows the disease significance of MF ontology. Transition metal ion binding is considered a significant function based on larger gene sets involving a total of 7 genes all of which contribute to RA pathogenesis (Supplementary Table S5, Fig. 3B). Supplementary Table S6 shows the significant genes grouped by functional categories defined by high-level GO terms. The DEGs are enriched in small molecule binding, molecular function regulators, transporter activity, enzyme regulator activity, and DNA-binding transcription factors.

#### Cellular components (CC)

In CC, DEGs were enriched in 42 pathways (Supplementary Table S4). Among the top pathways sorted by fold enrichment, DEGs were significantly enriched in a secretory vesicle, secretory granule, vesicle lumen, cytoplasmic vesicle lumen, and secretory granule lumen (Fig. 2C). Correlation of the top 10 pathways is represented in the hierarchical clustering tree (Supplementary Table S5, Supplementary Fig. 1C). Secretory granule and secretory vesicle pathways represent larger gene sets, with more overlap and enriched gene sets. The bar plot shows the disease significance of CC ontology. Secretory granule and secretory vesicle are considered significant components based on larger gene sets involving a total of 8 genes, out of which 6 contribute to SLE pathogenesis (Supplementary Table S5, Fig. 3C). Supplementary Table S6 shows the significant genes grouped by functional categories defined by high-level GO terms. The DEGs are enriched in the extracellular region and extracellular space.

**Fig. 4:**
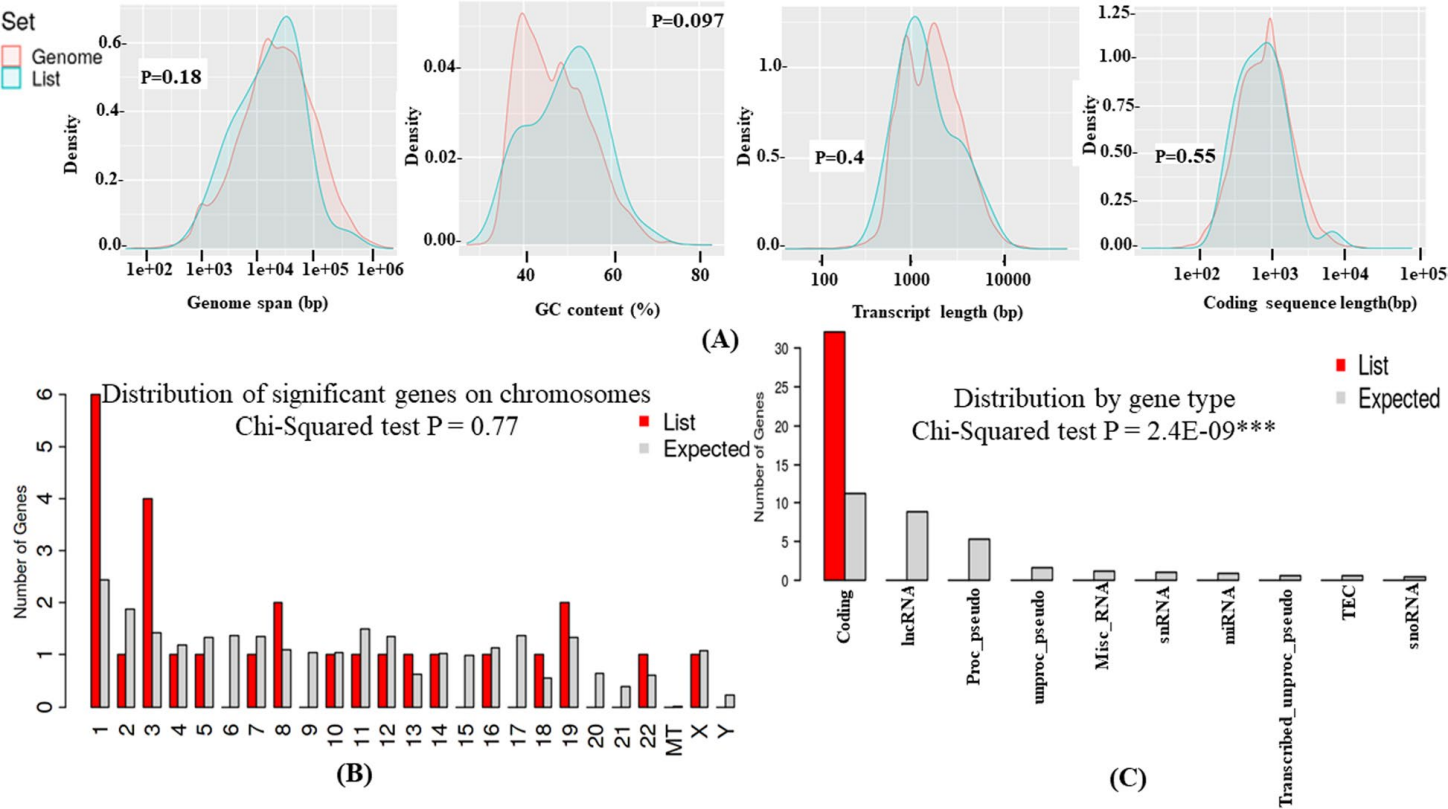
Chi-squared and Student’s *t*-tests analysis. **A** Distribution of the coding sequence length, transcript length, genome span, and GC content in significant DEGs versus other coding genes in the genome; **B** distribution of significant genes on chromosomes; and **C** distribution of significant genes by gene type. Analysis results have been provided in the supplemental information

A chi-squared and Student’s *t*-test analysis was performed to determine if significant genes have any special characteristic features by comparing them with other genes in the genome. As shown in Fig. 4A, the DEGs seem to have more genome span and transcript length than other genes in the genome. On the other hand, GC content and coding sequence length are comparatively low with reference to the genome gene sets. DEGs were significantly distributed mainly on chromosome 1 in autosomes and chromosome-X in allosomes. In contrast, no genes were found on 6, 9, 15, 17, 20, 21, and Y-chromosomes (Fig. 4B). Most of the DEGs identified are protein-coding genes (Fig. 4C and Supplementary Table S7).

### KEGG enrichment analysis of DEGs

KEGG enrichment analysis showed that significant genes were enriched in 46 pathways. The DEGs were enriched in pathways of endocytosis, phagosome, spliceosome, IL-17 signaling pathway, cell cycle, osteoclast differentiation, and so on (Supplementary Table S8). These pathways mainly involve immune cell activation, innate immunity, acquired immunity, and host defense against bacterial, fungal, and viral infections.

### Network topology-based analysis (NTA)

**Fig. 5:**
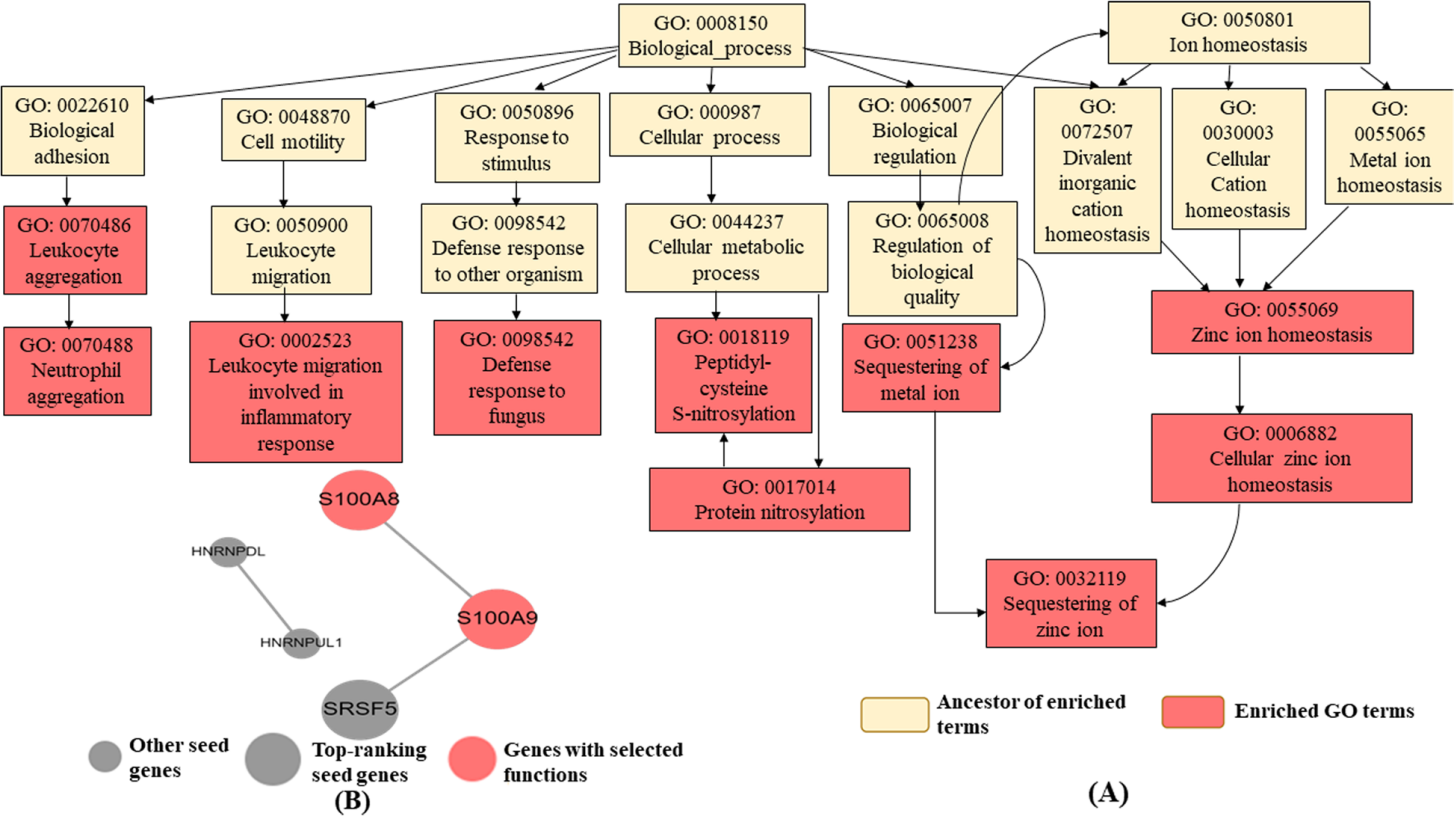
Network topology-based analysis using the PPI BioGRID network for expansion. **A** Construction of top 10 enriched gene ontology biological process terms. **B** Sub-network graph obtained from enrichment analysis. Gene ontology enrichment analysis of biological process terms is visualized as a dendrogram with the ten significantly enriched terms highlighted in red. The ancestors of these enriched terms are shown in light yellow. The sub-network graph contains five seeds (S100A8, S100A9, HNRNPDL, SRSF5, HNRNPUL1), among which calprotectin proteins (S100A8 and S100A9) are presented as neighbor seeds that are highlighted in all ten enriched GO terms

The network topology was derived from the significant DEGs using WebGestalt, comprising 32 input seeds, among which 31 were selected in the network. It highlighted 10 GO categories (Neutrophil aggregation, sequestering of zinc, leukocyte migration involved in the inflammatory response, leukocyte aggregation, protein nitrosylation, peptidyl-cysteine S-nitrosylation, sequestering of metal ions, defense response to fungus, cellular zinc ion homeostasis, and zinc ion homeostasis) as the top 10 enriched GO pathways for biological process terms (Supplementary table S9, Fig. 5A). The sub-network graph contains five seeds (S100A8, S100A9, HNRNPDL, SRSF5, HNRNPUL1), among which calprotectin proteins (S100A8 and S100A9) are presented as neighbor seeds that are highlighted in all ten enriched GO categories (Fig. 5B). Literature sources suggest that these proteins are regarded as proinflammatory cytokines and play a crucial role in inflammation. These are also found to be linked with cancer [42].

### Construction of protein–protein interaction (PPI) network and identification of candidate hub genes

**Fig. 6:**
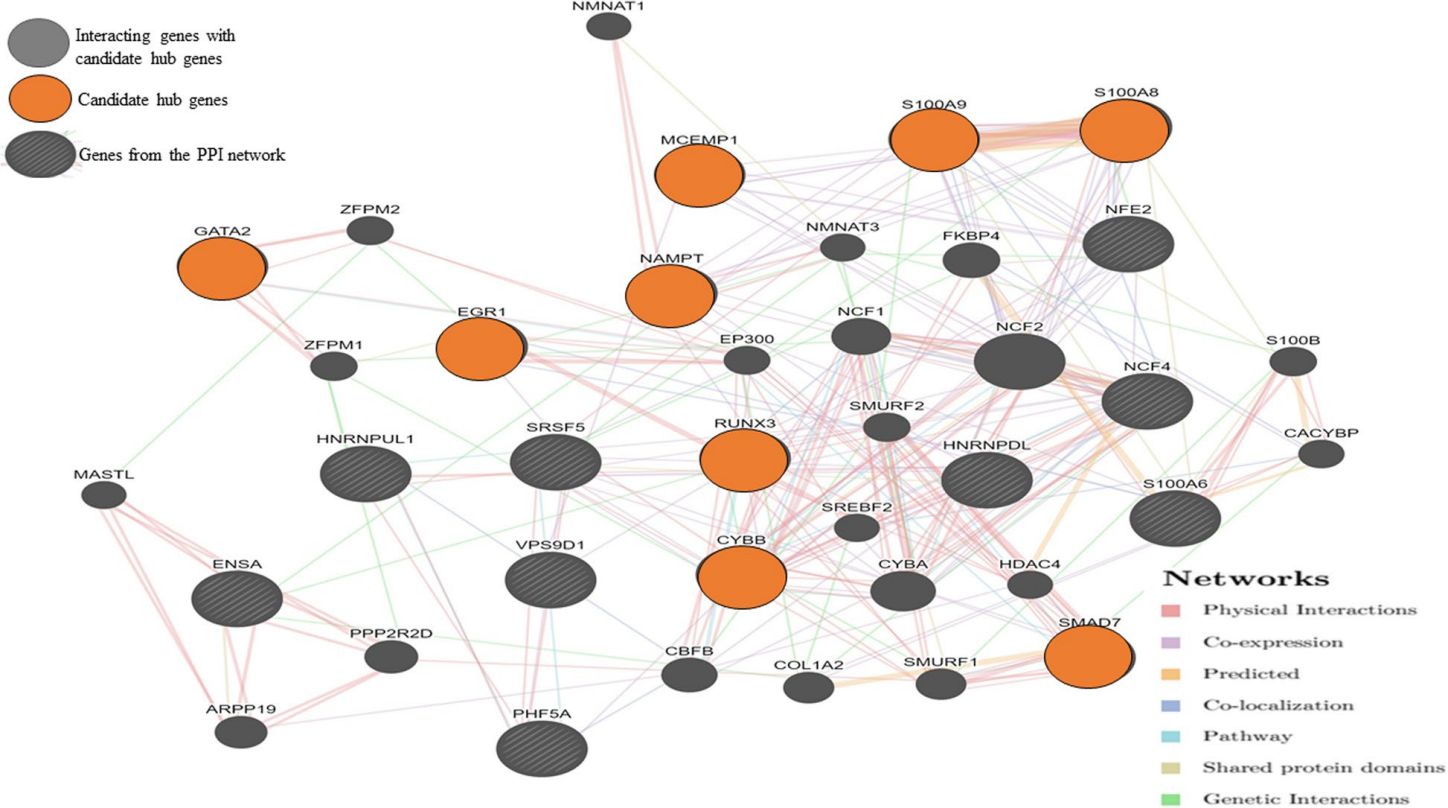
The gene interaction network of 9 candidate hub genes shows 20 related genes and 331 total links. 18 interacted nodes from the PPI network whose combined score > 0.4 were visualized and candidate hub genes were selected based on their interaction and involvement in both cell and leukocyte activation pathways, a key process in pathology associated with the autoimmune response

The network of significant DEGs showed possible interactions between the genes (Supplementary Fig. 2). The PPI enrichment *p*-value is found to be 8.04e − 07. The proteins interact more than expected for a random set of proteins of the same size and degree of distribution drawn from the genome. Such an enrichment indicates that the proteins are at least partially biologically connected. Out of 32, 18 nodes that had interactions were visualized using the GeneMANIA Cytoscape plugin command line tool (Fig. 6).

The network shows 20 related genes with 38 total genes and 331 total links based on protein–protein interaction data collected from BioGRID and PathwayCommons, genetic interaction data, shared protein domains, co-localization, pathway data, and predicted functional relationships between the genes [39]. S100A8 shows a shared protein domain with the S100A9 protein. These are myeloid-related proteins that activate the innate immune system to alert monocytes and macrophages to mediate inflammation [43]. The network also shows genetic interactions between RUNX3, CYBB, ENSA, and NAMPT. SMURF1 (Smad ubiquitination regulatory factor 1) has a genetic interaction with CYBB and NMNAT3 (Nicotinamide Nucleotide Adenylyltransferase 3) has a genetic interaction with EGR1. SMURF1 is a protein-coding gene that is associated with inflammatory bowel disease and acts as a negative regulator of the BMP (Bone morphogenetic protein) signaling pathway (regulation of cell motility, cell signaling, and cell polarity) [44]. NMNAT3 is reported to play a neuroprotective role as a molecular chaperone [45]. ENSA (Endosulfine Alpha) is also a protein-coding gene that plays a very important role in modulating insulin secretion through the interaction with KATP (ATP-sensitive potassium channel) channel, and this gene has been proposed as a candidate gene for type 2 diabetes [46, 47]. Also, S100A8, S100A9, MCEMP1, NAMPT, RUNX3, and SMAD7 are found to be co-expressed in the network (Supplementary Table S10). GO analysis revealed that these genes were involved in cell and leukocyte activation in Supplementary Table S5. Based on the network constructed, these genes were selected as candidate hub genes—EGR1, RUNX3, SMAD7, NAMPT, S100A9, S100A8, CYBB, GATA2, and MCEMP1. These are reported to be involved in leukocyte and cell activation, IL-17 signaling pathway, AGE-RAGE signaling pathway in diabetic complications, etc., (Supplementary Table S10). Thus, these candidate hub genes are linked to immune-related pathways, especially in AD pathogenesis.

### Validation of candidate hub genes and their significance

**Fig. 7:**
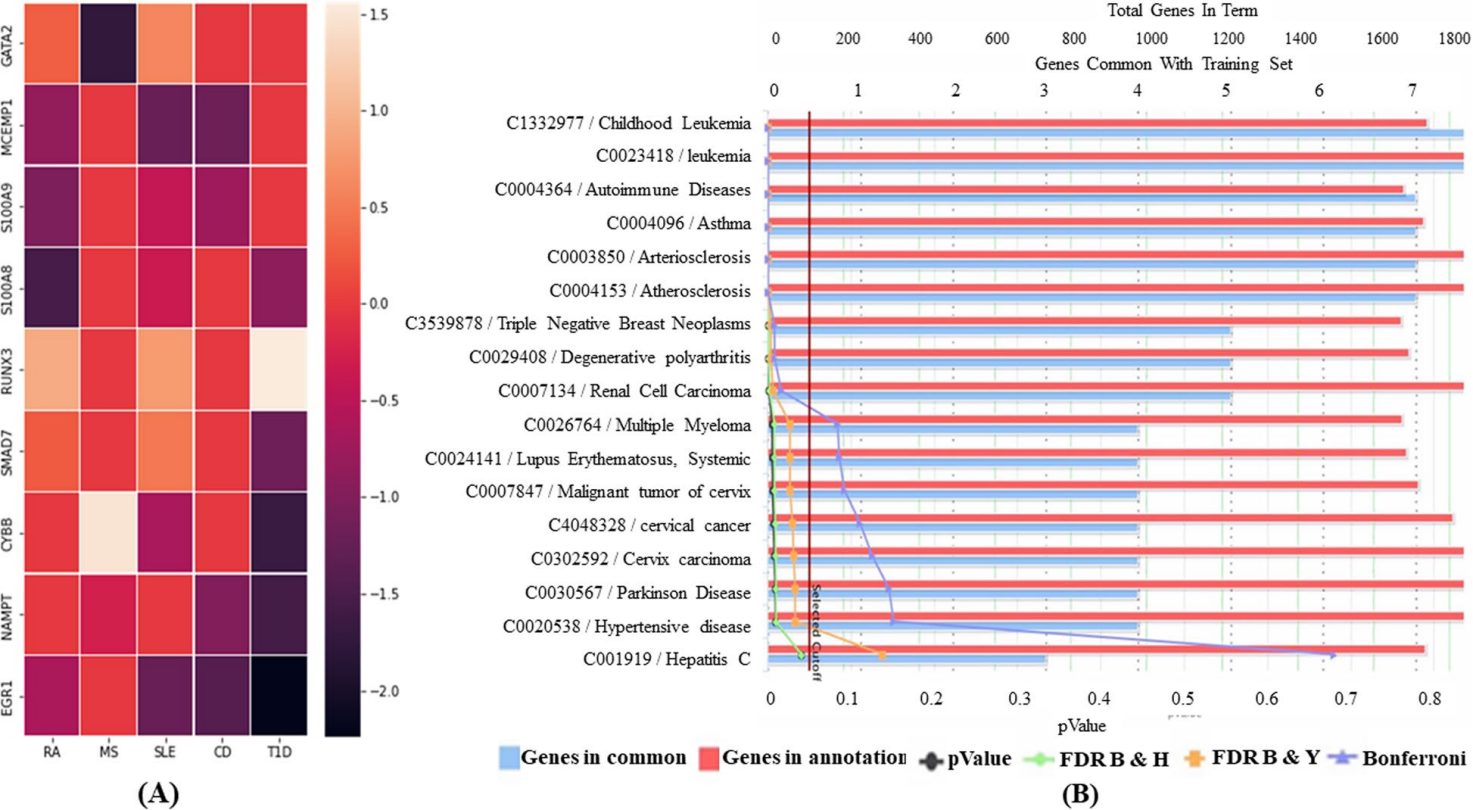
**A** Correlation heatmap of the candidate hub genes. **B** Significance of candidate hub genes in contributing to various other disorders with increased frequency in autoimmune diseases

The hub genes were validated to get significant results. EGR1, S100A8, S100A9, NAMPT, and MCEMP1 are downregulated in expression, whereas GATA2 is upregulated in RA, SLE, and downregulated in MS datasets. On the other hand, SMAD7 is upregulated in RA, and SLE and downregulated in T1D. In contrast, CYBB is downregulated in T1D, and SLE and upregulated only in MS. Notably, RUNX3 was upregulated in all ADs (RA, MS, and T1D) (Fig. 7A). The significance of the candidate hub genes was further validated from the literature sources. Also, its contribution to various other disorders was analyzed using ToppGene Suite (http://toppgene.cchmc.org) and found all these genes were primarily involved in leukemia disorders, arteriosclerosis, atherosclerosis, asthma, Parkinson’s diseases, apart from autoimmunity (Fig. 7B and Supplementary table S11).

### Chemical—gene interaction network analysis

**Fig. 8:**
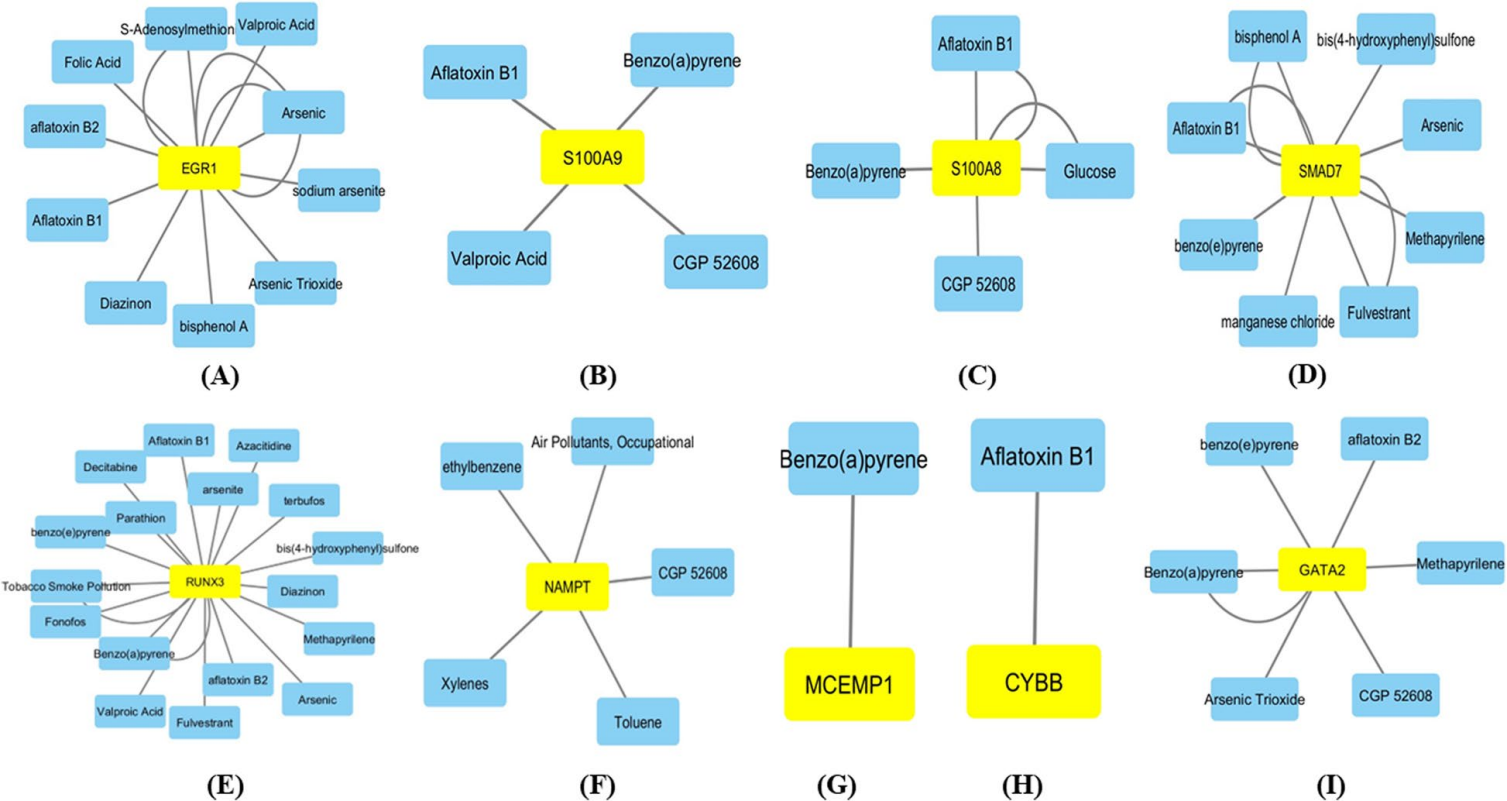
Chemical-gene interactions network with therapeutic drugs and chemical compounds and nine diagnostic genes was constructed using the CTD database. **A**–**I** The interaction between existing therapeutic drugs and the diagnostic genes. **A** EGR1. **B** S100A9. **C** S100A8. **D** SMAD7. **E** RUNX3. **F** NAMPT. **G** MCEMP1. **H** CYBB. **I** GATA2. CTD, comparative toxicogenomics database

The chemical or therapeutic compounds interacting with the diagnostic genes EGR1, RUNX3, SMAD7, NAMPT, S100A9, S100A8, CYBB, GATA2, and MCEMP1 were analyzed with the CTD database. The interaction network was created and visualized using Cytoscape (Fig. 8). The network showed that multiple chemicals could affect the expression of these nine diagnostic genes. EGR1 shows interaction with several chemicals, such as aflatoxin B1, aflatoxin B2, arsenic, folic acid, S-adenosylmethionine, arsenic trioxide, sodium arsenite, valproic acid, bisphenol A, and diazinon. These interactions result in decreased or increased methylation of EGR1 introns, promotors, and genes, thereby affecting its expression [48–55]. RUNX3 shows interaction with aflatoxin B1, aflatoxin B2, arsenic, arsenite, benzo(a)pyrene, benzo(e)pyrene, bis(4-hydroxyphenyl)sulfone co-treated with fulvestrant, tobocco smoke pollution, methapyrilene, decitabine, fonofos, etc., in which all of these genes result in increased or decreased methylation of RUNX3 gene, promoter, exons, and introns affecting its expression [48, 54–68]. SMAD7 shows its interaction with aflatoxin B1, arsenic, benzo(e)pyrene, bis(4-hydroxyphenyl)sulfone co-treated with fulvestrant, bisphenol A co-treated with fulvestrant, bisphenol A, manganese chloride and methapyrilene resulting in the increased or decreased methylation of the SMAD7 gene and intron affecting its expression [48, 51, 56, 58, 69]. NAMPT interaction with air pollutants, occupational chemicals, CGP 52608, toluene, ethylbenzene, and xylenes affects the gene’s expression by increasing the NAMPT gene’s methylation [70, 71]. S100A8 interacts with aflatoxin B1, benzo(a)pyrene, CGP 52608, and glucose. Literature sources suggest that these interactions promote the reaction by increasing the methylation of the S100A8 gene, intron, and promoter, which affects its expression level in the signaling cascade [48, 57, 61, 71, 72]. S100A9 shows interaction with aflatoxin B1, benzo(a)pyrene, CGP 52608, and valproic acid, affecting its gene expression by a biochemical process called methylation and also promoting the reaction [57, 61, 68, 71]. CYBB is found to interact with only one molecular compound aflatoxin B1 whose interaction results in decreased methylation of the CYBB gene [57]. GATA2 shows its interaction with six molecular compounds such as aflatoxin B2, arsenic trioxide, benzo(a)pyrene, benzo(e)pyrene, CGP 52608, and methapyrilene which decreases the methylation of GATA2 exon, affects the methylation of GATA2 promoter and intron, also promotes the reaction by increased methylation of GATA2 exon upon its interaction with these compounds [48, 50, 61, 71]. The interaction of MCEMP1 is found with only one molecular compound, benzo(a)pyrene whose interaction affects the methylation of MCEMP1 promoter [61] (Supplementary Table S12).

## Discussion

Autoimmune disorders (ADs) are chronic conditions that result from failure or breakdown of immunological tolerance, resulting in the host immune system attacking its cells or tissues. There is no cure, and current treatment aims only to reduce the chronic symptoms and lower the level of the immune system [18, 19, 73]. Present advances in our understanding of the human genome, gene expression, and genetic architecture are paving the way for new opportunities to understand the root cause of this disorder and advance the vision of developing improved strategies for the diagnosis and prognosis of autoimmune disorders. 19 transcriptome datasets of whole blood/peripheral blood cell samples with five different autoimmune disorders (Rheumatoid arthritis, multiple sclerosis, systemic lupus erythematosus, Crohn’s disease, and type 1 diabetes) were retrieved from the GEO database. A comparison of the differentially expressed genes from five different datasets representing autoimmune disorders from whole blood samples shows that 32 genes are common in more than two autoimmune disorders and are involved in 46 pathways such as Endocytosis, phagosome, IL-17 signaling, and so on. The gene–gene interaction network of the differentially expressed genes indicates the involvement of the transforming growth factor beta receptor signaling pathway, BMP (Bone morphogenetic proteins) signaling pathway, antigen processing and presentation, T-cell differentiation, leukocyte aggregation, and lymphocyte differentiation that are linked to autoimmune disorders. Apart from gene–gene interaction, environmental factors such as infectious agents, ultraviolet light, chemicals, environmental pollutants, smoking, and diet are linked with multiple genes in triggering autoimmune responses [9–13]. Our current study identifies some biomarkers and top differentially expressed genes that directly link to environmental factors. NAMPT, one of the biomarker genes identified in the study, regulates the activity of NAD-consuming enzymes such as sirtuins and influences various metabolic stress responses involved in inflammation [74]. Beta-2 adrenergic receptor (ADRB2), the top differentially expressed gene in systemic lupus erythematosus, is a G-protein coupled receptor superfamily member. Different polymorphic forms, point mutations, and downregulation of this gene are involved in the pathogenesis of obesity and are also involved in nocturnal asthma, type 2 diabetes, and cardiovascular disease pathways [75].

We propose an integrated gene expression analysis to identify marker genes in the present study. A total of 9 biomarker genes, EGR1, RUNX3, SMAD7, NAMPT, S100A9, S100A8, CYBB, GATA2, and MCEMP1, were identified as candidate hub genes that are involved in the AD pathogenesis. All genes were downregulated in expression except RUNX3, which was upregulated in type 1 diabetes, and CYBB in multiple sclerosis.

As a member of the EGR family of C2H2-type zinc-finger proteins, Early Growth Response 1 (EGR1) is a transcriptional regulator involving numerous physiological processes such as synaptic plasticity, wound repair, inflammation, and differentiation. It is expressed in the thymus, cartilage, bones, muscles, endothelium, and central and peripheral nervous system [76]. Numerous studies on this gene at transcriptional and protein levels show it is involved in both cell proliferation and apoptosis [77, 78]. It regulates the expression of proteins such as IL1B (interleukin-1 beta) and CXCL2 (C-X-C motif chemokine ligand 2) involved in the inflammatory process and the development of tissue damage after ischemia. It also mediates hypoxia [79, 80] and has tumor suppressor properties [81]. Apart from these functions, EGR1 plays a crucial role in the immune system by determining the differentiation pathway of myeloid cell precursors [82]. It is an important transcription factor in multiple sclerosis, confirming its role in brain plasticity and many other neuropsychiatric disorders [83, 84]. Thus, EGR1 is one of the biomarkers in autoimmune disorders and is confirmed in our study.

Belonging to the family of runt domains, RUNX3 is an essential transcriptional factor and a key regulator for lineage-specific gene expression. Expression of RUNX3 is predominant in all hematopoietic origin of cells such as the thymus, spleen, myeloid, peripheral blood, and B- and T-cell lineages within the bone marrow [85, 86]. Any alteration in this expression level results in various human diseases [87]. The expression of RUNX3 is crucial for the development of CD8-lineage T lymphocytes. Failure results in an inadequate response towards antigens, contributing to the origin of autoimmune diseases [88, 89]. Many studies have confirmed the role of the RUNX3 gene in the pathogenesis of rheumatoid arthritis, lupus, and psoriasis [89]. Another interesting finding by Ofer Fainaru and his colleagues reported that lack of RUNX expression in a mouse model resulted in eosinophilic lung inflammation. They found the expression of RUNX3 in mature dendritic cells of the mouse model. These dendritic cells mediate RUNX3 response to the TGF β (transforming growth factor β) signaling pathway. When this gene was knocked out in the mouse model, it resulted in rapid maturation of dendritic cells and increased stimulation of T-cells. These abnormalities in dendritic cells result in the defect of the primary immune system leading to eosinophilic lung inflammation. Thus, this study confirms that RUNX3 is the critical regulator of thymopoiesis [90]. Apart from these functions, RUNX3 is also a significant tumor suppressor gene. Studies report that its inactivation results in breast cancer [91] and gastric cancer [92].

SMAD7 gene is also known as mothers against decapentaplegic homolog 7 (MADH7), a gene from the I-Smads (inhibitory Smads) family*.* The protein product of this gene inhibits the TGF β (transforming growth factor β) signaling pathway. The TGF β pathway is very crucial in the pathological process of various disorders. Activation of the TGF β receptor results in the release of SMAD7 from the nucleus into the cytoplasm, where it inhibits the phosphorylation of Smad2/3 or induces the degradation of TGF-β receptor I and Smad2/3, thereby disrupting its joint partner Smad4. Thus, SMAD7 is a negative regulator of the TGF-β signaling pathway [93]. Also, the DNA binding activity of NF-κB is inhibited if SMAD7 is overexpressed [94]. This is the key mechanism involved in a lot of inflammatory diseases. So, inhibition of SMAD7 results in activation of NF-κB and TGF-β signaling pathways, making a way to prevent and treat various disorders [95]. Furthermore, the amount or level of its expression can also be used as a marker in various diseases. SMAD7 is also involved in immunomodulatory functions and has both prophylactic and therapeutic potential. Apart from inflammation, these proteins are also involved in cancer, kidney disease, and many other diseases affecting migration, fibrosis, proliferation, and apoptosis. Therefore, SMAD7 is an attractive target for many disorders, including immune-related conditions [95].

Nicotinamide phosphoribosyl transferase (NAMPT) is also known as visfatin [96]. It is an enzyme encoded by the NAMPT gene. The intracellular form of this protein (iNAMPT) is the rate-limiting enzyme in the nicotinamide adenine dinucleotide (NAD +) salvage pathway that converts nicotinamide to nicotinamide mononucleotide (NMN), which is responsible for most of the NAD + formation in mammals [97]. NAD + is involved in more than 500 enzymatic reactions, including redox reactions. Recent publications suggest the importance of NAD + as if its concentration or level is increased in diseased or old animals, it improves their health and also increases their lifespan [98]. Rajman et al. have confirmed the importance of NAD + in many biological processes such as mitochondrial function and metabolism, the 45 circadian rhythms, immune response and inflammation, DNA repair, cell division, 46 protein–protein signaling, chromatin remodeling, and epigenetics, confirming it is a molecule of life in mammals [99]. Apart from these functions, NAMPT also connects the sirtuin (SIRT) signaling of NAD-dependent against various stress responses [100]. On the other hand, extracellular NAMPT (eNAMPT) is reported to be involved in the pancreatic β-cell function. Many studies supported its involvement by providing indirect evidence for the connection between eNAMPT and insulin signaling. Thus, NAMPT could be an effective therapeutic target for preventing and treating metabolic disorders, including obesity and type 2 diabetes mellitus, inflammation, and cancer [74].

S100A8 and S100A9 are also known as myeloid-related protein 8 (MRP8) and myeloid-related protein 14 (MRP14), belonging to the family of S100 [101]. They are found as heterodimers in the cytoplasm of neutrophils and monocytes. They are calcium-binding proteins whose expression is constitutive in the cells of myeloid origin [102]. During pathological conditions, our immune system gets activated, releasing many cell-activating factors such as chemokines, chemokine receptors, alarmins, and their respective pattern recognition receptors [103]. One such alarmin is S100A8 and S100A9, released from neutrophils and monocytes that bind to two pattern recognition receptors such as Toll-like receptor 4 (TLR4) and Receptor of Advanced Glycation Endproducts (RAGE). This activates the innate immune system to alert monocytes and macrophages to mediate inflammation [43]. In 2004, Foell and his colleagues found the over-expression of S100 proteins, particularly S100A8, S100A9, and S100A12, at the site of inflammation in Rheumatoid arthritis, chronic inflammatory lung, and bowel disease. So, they confirmed that these S100 proteins could serve as laboratory markers in many inflammatory pathologies [104]. Recent studies on COVID patients also report that these proteins induce aggressive inflammation in severe acute respiratory syndrome coronavirus 2 (SARS-CoV-2) infection, and it can be used as a novel biomarker [105]. This evidence supports that calprotectin proteins (S100A8/A9) are proinflammatory cytokines and could serve as an interesting drug target by blocking its activation in autoimmune disorders.

NADPH oxidase 2, abbreviated as Nox2, is also known as cytochrome b (558) subunit beta or cytochrome b-245 heavy chain. NOX2 is called CYBB or gp91phox (encoded by the CYBB gene)**.** It is a multicomponent enzyme complex consisting of 5 subunits such as CYBA (cytochrome B-245 alpha chain), CYBB (cytochrome B-245 beta chain), NCF1 (neutrophil cytosolic factor 1), NCF2 (neutrophil cytosolic factor 2), and NCF4 (neutrophil cytosolic factor 4) [106, 107]. Its expression is found in myeloid cells such as monocytes, macrophages, and neutrophilic granulocytes. The primary and known function of the NOX2 gene is the generation of toxic derivatives of oxygen (reactive oxygen species (ROS)). Myeloid cells produce NOX2-derived ROS as part of the innate immune defense against bacteria and other microorganisms. When the level of ROS is increased, it results in oxidative stress. This results in many pathologies, including autoimmune disorders [107]. Giulia Cardamone and his colleagues in the year 2018 found the altered expression of the CYBB gene resulting in the demyelination and axonal injury in both multiple sclerosis (MS) and experimental autoimmune encephalomyelitis in the murine model [108]. They found that the differential expression of the CYBB gene resulted in the excessive production of ROS. This activates nuclear transcription factor kappa B along with other specific transcription factors, and on the other hand, it also activates matrix metalloproteinases. These events activate proinflammatory cytokines and particular genes related to MS (genes are upregulated), resulting in disease progression [109–115]. On the other hand, decreased ROS levels also contribute to autoimmunity [108]. Mutation of the CYBB gene results in the most common form of the chronic granulomatous disease (CGD), which is X-linked recessive. Mostly males are affected by this primary immunodeficiency disorder. The people affected by this disorder have impaired or no production of phagocyte NADPH oxidase because neutrophils can phagocytize bacteria but cannot kill them in the phagocytic vacuoles [116]. It is also reported that one-third to one-half of children with CGD develop gastrointestinal inflammation, usually Crohn’s disease. Some studies have listed CYBB as a gene related to inflammation associated with kidney disease in type 1 diabetes [116, 117]. Thus, CYBB could be an effective therapeutic target for the prevention and treatment of immunological pathologies.

GATA binding protein 2 (GATA2) is one of the 6 GATA binding factors binding to the DNA motif GATA and other transcription factors via two zinc finger domains to regulate its expression at multiple levels [118, 119]. It is a key transcriptional regulator of hematopoiesis for the survival, self-renewal, development, and maintenance of a healthy stem cell pool [119]. The GATA2 gene encodes GATA2 protein. When particular genes are “turned on,” GATA2 protein directs the activity of many types of cells, including immune cells. Any mutation or impairment in the GATA2 gene results in GATA2 deficiency, presenting several distinct syndromes in the dendritic cell, monocyte, B, natural killer lymphoid deficiency, acute myeloid leukemia, and natural killer (NK) cell deficiency [119]. Although it plays a vital role in hematopoiesis, Rossella Menghini and her colleagues hypothesized that GATA2 activity is controlled by insulin during adipogenesis, linking metabolic homeostasis and inflammation. They found that the phosphorylation of GATA2 on serine 401 in a PI-3 K/Akt–dependent manner results in the development of adipocytes and no expression of inflammatory markers such as monocyte chemotactic protein-1 (MCP-1). They concluded their study that GATA2 can be a new target in preventing and treating obesity-related inflammation and its complications [120]. From all this evidence, it is clear that GATA2 can be a therapeutic biomarker in inflammation-related disorders.

Mast cell-expressed membrane protein, or MCEMP1, is the protein product of the MCEMP1 gene. It was found to be differentially expressed in mast cells. In the human defense system, there are many immune-related cells, among which mast cells are major immune effector cells against parasite infection. Activation of mast cells results in the release of many preformed secretory inflammatory mediators (histamine, tryptase, and neutrophil chemotactic factor), cytokines (TNFα, IL-4, IL-13, IL-5, IL-10), and chemokines [121–123]. Mast cells are essential in pathologic conditions such as asthma, allergic rhinitis, anaphylaxis, and atopic dermatitis. It is also involved in many cancer-related and inflammatory processes such as gastric cancer, rheumatoid arthritis, and multiple sclerosis [124–129]. The mast cell concentration is higher in some inflammatory disease conditions, such as inflammatory bowel disease [130]. Thus, the protein product of this gene is a single-pass transmembrane protein involved in regulating mast cell differentiation or immune responses. Hence, MCEMP1, based upon its concentration or level in many immune-related and inflammatory disorders, can also be used as an attractive biomarker for the diagnosis, prevention, and treatment of autoimmune disorders.

## Conclusion

To conclude, integrated gene expression analysis was employed on 5 different autoimmune disorders to find shared genes and their pathways for the prevention and treatment of autoimmune disorders. This analysis has identified nine biomarker genes, EGR1, RUNX3, SMAD7, NAMPT, S100A9, S100A8, CYBB, GATA2, and MCEMP1, and their role in the early prediction of immune-related disorders, especially in a lot of inflammatory and autoimmune pathologies. Also, this study strengthens previous literature sources of confirming these biomarkers linked to causing inflammation and autoimmune disorders. The treatment options that remain today are limited and have no cure due to their complexity. Present advances in our understanding of the human genome, gene expression, and genetic architecture are making a path for new opportunities to understand the root causes of this disorder and advance the vision of developing improved strategies for the prevention and treatment of autoimmune disorders in the near future.

### Supplementary Information

**Additional file 1.**

**Additional file 2.**

**Additional file 3.**

**Additional file 4.**

**Additional file 5.**

**Additional file 6. **

**Additional file 7: Supplementary Fig. 1.**Hierarchical clustering tree of 32 DEGs in (A) Biological processes (B) Molecular functions and (C) Cellular components. Pathways with shared genes are clustered together. Bigger dots indicate more significant p-values. Detailed GO analysis has been provided in the supplemental information.**Supplementary Fig. 2.**Protein-protein interaction (PPI) network constructed using the STRING database gave a total of 32 nodes; 20 edges with an average node degree of 1.25; out of which 18 nodes exhibit known and predicted interaction and a PPI enrichment p-value of 8.04e−07.

**Additional file 8.**

**Additional file 9.**

**Additional file 10.**

**Additional file 11.**

**Additional file 12.**

**Additional file 13.**

**Additional file 14.**

## Data Availability

All Datasets included in this study can be downloaded from the Gene Expression Omnibus database (GEO, https://www.ncbi.nlm.nih.gov/geo/).
