# Supplementary material for: Identification of common genetic factors and immune-related pathways associating more than two autoimmune disorders: implications on risk, diagnosis, and treatment
Source: Genomics Inform. 2024 Jul 2;22:10. doi: 10.1186/s44342-024-00004-5 (PMC11221123; doi:10.1186/s44342-024-00004-5)
Supplement: Supplementary file 2 — Additional file 2. [file 44342_2024_4_MOESM2_ESM.docx]

**Supplementary Table S1 : Summary Information of Autoimmune disorders included in our study**

| **AUTOIMMUNE DISORDERS** | **GEO ACCESSION** | **PUBLIC DATE** | **PLATFORM** | **TISSUE** | **SAMPLE COUNT**  **(NC: AD)** |
| --- | --- | --- | --- | --- | --- |
| Rheumatoid arthritis | GSE7524 | Jul 07, 2007 | [GPL96[HG-U133A] Affymetrix Human Genome U133A Array](https://www.ncbi.nlm.nih.gov/geo/query/acc.cgi?acc=GPL96) | Whole blood | 2:2 |
|  | GSE93272 | Jul16,  2008 | GPL570 [HG-U133_Plus_2] Affymetrix Human Genome  U133 Plus 2.0 Array | Whole blood | 35:45 |
|  | GSE15573 | Sep03,  2009 | [GPL6102 [Illumina human-6 v2.0 expression bead chip](https://www.ncbi.nlm.nih.gov/geo/query/acc.cgi?acc=GPL6102)] | Peripheral blood mononuclear cells | 15:18 |
|  | GSE17755 | Aug 21, 2010 | [GPL1291 [Hitachisoft AceGene Human Oligo Chip 30K 1 Chip Version](https://www.ncbi.nlm.nih.gov/geo/query/acc.cgi?acc=GPL1291)] | peripheral blood cells | 53:112 |
|  | GSE134087 | Jun 10, 2020 | [GPL16956 [Agilent-045997 Arraystar human lncRNA microarray V3 (Probe Name Version)](https://www.ncbi.nlm.nih.gov/geo/query/acc.cgi?acc=GPL16956) | peripheral blood | 2:2 |
| Multiple Sclerosis | GSE17048 | Apr 02, 2010 | GPL6947[llumina HumanHT-12 V3.0 expression beadchip] | Whole blood | 45:99 |
|  | GSE21942 | Jul 01, 2011 | GPL570[HG-U133_Plus_2] Affymetrix Human Genome U133 Plus 2.0 Array | Peripheral blood mononuclear cells | 15:12 |
|  | GSE26484 | Jun 01, 2011 | GPL570[HG-U133_Plus_2] Affymetrix Human Genome U133 Plus 2.0 Array | Peripheral blood cells | 4:6 |
|  | GSE141804 | Dec 11, 2020 | GPL96[HG-U133A] Affymetrix Human Genome U133A Array  and  GPL571 [HG-U133A_2] Affymetrix Human Genome U133A 2.0 Array | peripheral blood mononuclear cells | 10:17 |
| Systemic lupus erythematosus | GSE17755 | Aug 21, 2010 | [GPL1291Hitachisoft AceGene Human Oligo Chip 30K 1 Chip Version](https://www.ncbi.nlm.nih.gov/geo/query/acc.cgi?acc=GPL1291) | Peripheral blood cells | 53:22 |
|  | GSE30153 | Jun 23, 2011 | [GPL570[HG-U133_Plus_2] Affymetrix Human Genome U133 Plus 2.0 Array](https://www.ncbi.nlm.nih.gov/geo/query/acc.cgi?acc=GPL570) | Peripheral blood B cells | 9:17 |
|  | GSE81622 | Jun 01, 2016 | [GPL10558I Illumina HumanHT-12 V4.0 expression beadchip](https://www.ncbi.nlm.nih.gov/geo/query/acc.cgi?acc=GPL10558) | Peripheral blood mononuclear cells | 25:30 |
|  | GSE72326 | Jan 02, 2022 | [GPL10558Illumina HumanHT-12 V4.0 expression beadchip](https://www.ncbi.nlm.nih.gov/geo/query/acc.cgi?acc=GPL10558) | Whole Blood | 20:157 |
| Diabetes | GSE11907 | Jun 26, 2008 | GPL96 [HG-U133A] Affymetrix Human Genome U133A Array  and  GPL97 [HG-U133B] Affymetrix Human Genome U133B Array | Peripheral-blood mononuclear cell | 12:20 |
|  | GSE142153 | Dec 01, 2020 | [GPL6480Agilent-014850 Whole Human Genome Microarray 4x44K G4112F (Probe Name version)](https://www.ncbi.nlm.nih.gov/geo/query/acc.cgi?acc=GPL6480) | Peripheral blood mononuclear cells | 10:23 |
|  | GSE168437 | Mar 23, 2021 | [GPL22517[OElncRNAs520855F] Affymetrix Human OElncRNAs520855F Array [probe set (exon) version]](https://www.ncbi.nlm.nih.gov/geo/query/acc.cgi?acc=GPL22517) | Peripheral blood mononuclear cells | 2:2 |
| Crohn’s Disease | GSE119600 | May 01, 2019 | GPL10558 Illumina HumanHT-12 V4.0 expression beadchip | whole blood | 47:95 |
|  | GSE126124 | Oct 29, 2019 | [GPL6244[HuGene-1_0-st] Affymetrix Human Gene 1.0 ST Array [transcript (gene) version]](https://www.ncbi.nlm.nih.gov/geo/query/acc.cgi?acc=GPL6244) | blood | 32:39 |
|  | GSE3365 | Sep 26, 2005 | [GPL96](https://www.ncbi.nlm.nih.gov/geo/query/acc.cgi?acc=GPL96) [HG-U133A] Affymetrix Human Genome U133A Array | Peripheral blood mononuclear cells | 42:59 |
