# Supplementary material for: Identification of common genetic factors and immune-related pathways associating more than two autoimmune disorders: implications on risk, diagnosis, and treatment
Source: Genomics Inform. 2024 Jul 2;22:10. doi: 10.1186/s44342-024-00004-5 (PMC11221123; doi:10.1186/s44342-024-00004-5)
Supplement: Supplementary file 7 — Additional file 7: Supplementary Fig. 1.Hierarchical clustering tree of 32 DEGs in (A) Biological processes (B) Molecular functions and (C) Cellular components. Pathways with shared genes are clustered together. Bigger dots indicate more significant p-values. Detailed GO analysis has been provided in the supplemental information.Supplementary Fig. 2.Protein-protein interaction (PPI) network constructed using the STRING database gave a total of 32 nodes; 20 edges with an average node degree of 1.25; out of which 18 nodes exhibit known and predicted interaction and a PPI enrichment p-value of 8.04e−07. [file 44342_2024_4_MOESM7_ESM.docx]

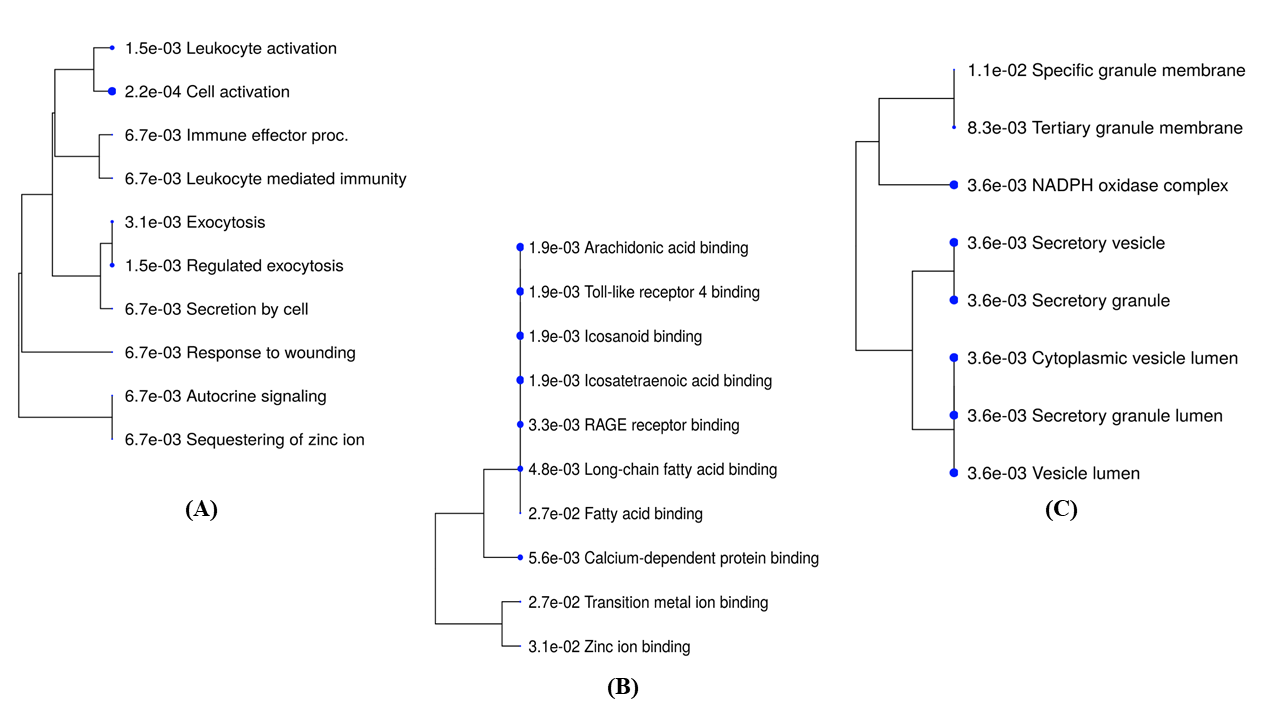
**Supplementary Fig. (1).** Hierarchical clustering tree of 32 DEGs in **(A)** Biological processes **(B)** Molecular functions and **(C)** Cellular components. Pathways with shared genes are clustered together. Bigger dots indicate more significant P-values. Detailed GO analysis has been provided in the supplemental information.


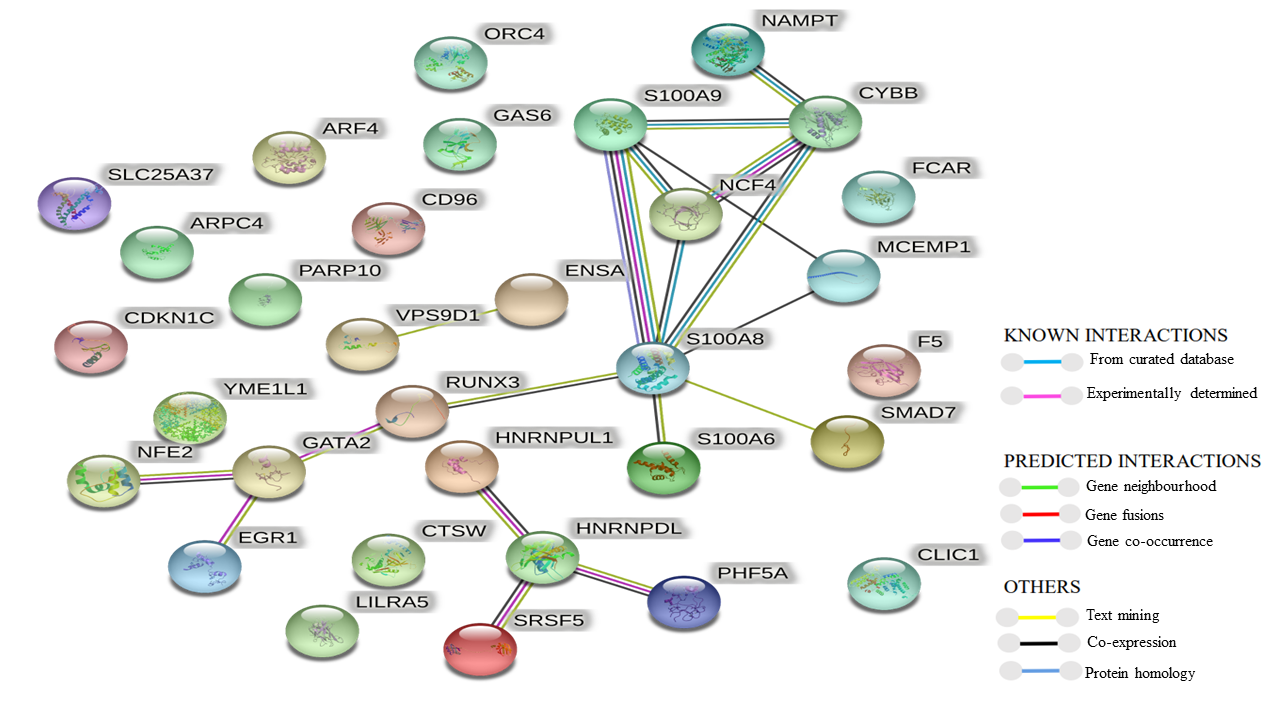
**Supplementary Fig. (2).** Protein-protein interaction (PPI) network constructed using the STRING database gave a total of 32 nodes; 20 edges with an average node degree of 1.25; out of which 18 nodes exhibit known and predicted interaction and a PPI enrichment p-value of 8.04e-07.
